# Hidden altruism in a real-world setting

**DOI:** 10.1098/rsbl.2013.0884

**Published:** 2014-01

**Authors:** N. J. Raihani

**Affiliations:** Department of Genetics, Evolution and Environment, University College London, London WC1E 6BT, UK

**Keywords:** cooperation, altruism, charity, reputation, anonymous

## Abstract

Concerns for reputation can promote cooperative behaviour. Individuals that behave cooperatively stand to benefit if they gain in influence, status or are more likely to be chosen as interaction partners by others. Most theoretical and empirical models of cooperation predict that image score will increase with cooperative contributions. Individuals are therefore expected to make higher contributions when observed by others and should opt to make contributions publicly rather than privately, particularly when contributions are higher than average. Here, however, I find the opposite effect. Using data from an online fundraising website, I show that donors are more likely to opt for anonymity when making extremely low and extremely high donations. Mid-range donations, on the other hand, are typically publicized. Recent work has shown that extremely generous individuals may be ostracized or punished by group members. The data presented here suggest that individuals may hide high donations to avoid these repercussions.

## 1. Introduction

Therefore when thou doest thine alms, do not sound a trumpet before thee, as the hypocrites do in the synagogues and in the streets, that they may have glory of men … . But when thou doest alms, let not thy left hand know what thy right hand doeth... (Matthew 6:2–4)

Cooperative behaviour often requires individuals to forego immediate benefits by investing to benefit interaction partners. One way that cooperative individuals might benefit from such an investment is through an improved reputation, which confers a higher status on the cooperative individual [1,2]. Individuals that have a good reputation are more likely to elicit investments from others (‘indirect reciprocity’) [1,3,4] and are also preferred over non-cooperative individuals as interaction partners [5,6]. Philanthropy—or donating to charity—is an overtly cooperative behaviour. In the vast majority of cases, individuals sacrifice financial resources to benefit others that are less well off, without the prospect of reciprocity from these beneficiaries in the future. It is now well appreciated that a central motivating force for donations to charity is concern for reputation [7,8]. Donations to charities are typically higher when the donation is publicized rather than anonymous [9–12] and, given the choice, individuals prefer to donate publicly rather than anonymously [9].

Despite this fact, several public fundraising platforms now offer donors the possibility to conceal their identity or the size of their donation when making contributions [9]. If charitable donations serve partly to signal the donor's generosity, then individuals are not expected to select this option [7]. Indeed, anonymous donations to charity are typically thought to represent less than 2% of all charitable donations in any given context [7]. Why, then, might individuals choose to make anonymous donations to fundraising appeals? The vast majority of studies, theoretical and empirical, have assumed that cooperative behaviour and reputation are positively linked such that an increase in cooperative behaviour results in improved reputation and the associated benefits that high-status brings [3,4,13–16]. However, exaggerated displays of cooperation may sometimes carry social costs. For example, Muslim participants in a cooperation experiment were more likely to donate and donated higher average amounts to a charitable cause when donations were anonymous rather than publicized [17]. Although this finding was explained in terms of the religion's proscription of non-sincere (status-seeking) generosity, the possibility that hidden altruism might also be common in different cultures and also outside of religious contexts has been less well explored. There is suggestive evidence that individuals may generally pay social costs associated with extremely altruistic displays. Recent work has shown that individuals that cooperate by making high contributions to a public good may be shunned [18] or even punished [19,20], even though the punitive group members ostensibly benefit from the investment of the cooperator. It has been suggested that negative feelings towards high cooperators stem from the fact that these individuals violate group norms and establish undesirable standards of cooperation, which make the contributions of others look less impressive by comparison [18–20]. Individuals that are sensitive to the social costs of high investments may avoid these costs by concealing high investments from others. I test this idea using data from real-world donations to an online fundraising site.

## 2. Material and methods

Using a well-known online fundraising website (BMyCharity, www.bmycharity.com) in the UK, I collated contributions to 110 fundraising appeals for 36 different UK registered charities from 2007 to 2013. This resulted in 3945 donations for analysis. Appeals were selected at random from the site; appeals with fewer than 10 donors were not included. Data were analysed using R v. 3.0.1 (www.r-project.org). Each donation was scored as ‘individual’, if the contribution was from a named individual or a family and ‘collective’, if the donation was from an organization or a company. A total of 129 donations were made by organizations or companies. As the mean donation amount made by collectives was significantly higher than the mean donations made by individuals (£170.21 ± 28.6 versus £29.64 ± 1.13, respectively; two-sample *t*-test, *t*_1,132_ = −14.7, *p* < 0.001) all analyses were restricted to donations made by individuals (*n* = 3816).

Unlike some other fundraising websites, BMyCharity does not offer donors an option to conceal the donated amount, so all donations were visible, even if the donor identity was concealed. I ran a generalized linear-mixed model (GLMM) with binomial distribution of errors and a logit-link function to test the hypothesis that extremely low and extremely high donations would be more likely to be anonymous than average donations. The binary variable ‘anonymity’ (1 = anonymous donation; 0 = public donation) was set as the response term and ‘donation amount’ was set as the explanatory term. Donation amount was a three-level factor (‘low’, ‘medium’ and ‘high’). Low donations were in the lower quartile of all donations for that appeal; medium donations were between the lower and upper quartile and high donations were above the upper quartile for that appeal. The terms ‘charity id’ and ‘fundraiser id’ were included in the model as random terms to control for the effects of these repeated terms on the distribution of the data. All donations were made in UK pounds (£).

## 3. Results

**Figure 1.**
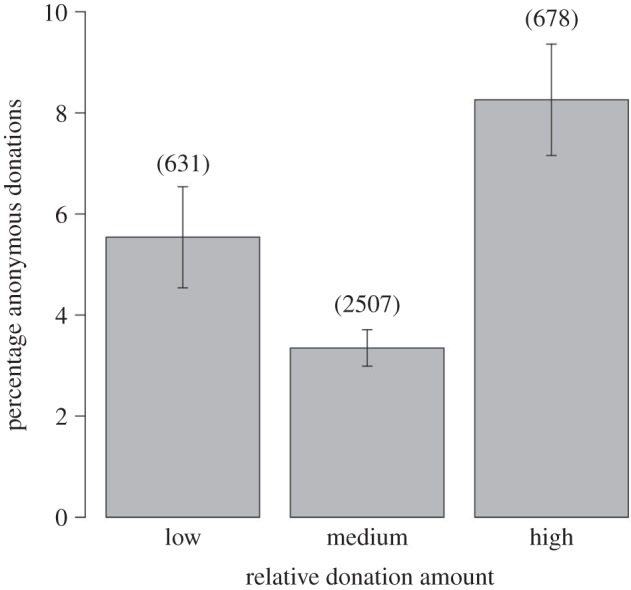
The mean (±s.e.) percentage of donations made anonymously according to the relative donation amount. Data from individual or family donations (not from companies or other collectives) only were used. Sample sizes are shown above each bar.

The median donation across all appeals was £20.00 (range = £10.00–25.00) with a mean of £29.64 ± 1.13. Of the 3816 donations made by individuals, 175 (4.6%) were made anonymously. Individuals were more likely to conceal their identity when making donations that were extremely low or extremely high, relative to other donations to that appeal (GLMM: *χ*^2^ = 26.7, d.f. = 2, *p* < 0.001; figure 1).

## 4. Discussion

These data suggest that people hide cooperative behaviour if that behaviour violates established norms and are consistent with previous findings on punishment in public goods games, which showed that both extremely low and extremely high contributors to the public good are likely to be ostracized or punished by group members [18–20]. When people are uncertain about the appropriate behaviour in a given context, they tend to be influenced by what others do in the same situation [21,22]. For example, an experiment designed to encourage the reuse of towels by hotel guests found that a sign which stated that the majority of guests reuse their towels was significantly more effective at encouraging towel reuse than an alternative sign highlighting the environmental benefits of towel reuse [23]. Social norms have also been shown to influence charitable giving [24,25]. For example, donations made by Zurich University students to a charitable organization increased when they were told that a relatively high proportion of fellow students also gave to the charity, as opposed to when they were told that a relatively low proportion of students donated [24]. It is likely that donors to fundraising websites experience a degree of uncertainty as to the appropriate amount to give to any given appeal and look to the previous donations made on that page to help to inform their decision. Donors who give amounts that violate the established norm in this context may opt for anonymity, as shown here.

While it is fairly intuitive that individuals who undercontribute to public goods will incur anger and possible punishment from fellow group members, it is less clear why people apparently dislike [18] and wish to punish overcontributors [19,20]. This is especially puzzling in the context of traditional public goods games, because the overcontributors confer benefits on others in the group while incurring personal costs. One possible explanation is that overcontributors establish undesirable behavioural standards in the group: the excessively high donations made by overcontributors may make the donations of the rest of the group look paltry in comparison. Given that there is suggestive evidence that humans are concerned with their reputation score relative to that of others [5,13,15,26] these high contributions may be perceived as a competitive, rather than cooperative, act and overly generous donors may risk social ostracism or punishment as a result. Indeed, a previous study has shown that charitable giving is at least partly motivated by competition. Subjects playing a sequential donation game increased their donations when the game was framed as a ‘generosity tournament’ rather than an ‘earnings tournament’ [27]. If overly generous contributions are viewed as competitive rather than cooperative acts, they may provoke negative responses from others.

There is another reason that high donations may be perceived negatively by others: if donation amount acts as a signal of wealth [28], individuals may use donation amount as a signal of success and skill and by implication to promote prestige [7,29]. This is similar to the explanation above but the difference here is that perceived wealth, rather than generosity is the mechanism for conferring prestige. Such showy behaviour could backfire, however, resulting in a negative assessment of the donor. For example, Fiske *et al*. [30] have shown that wealthy individuals are perceived not only as highly competent, but also as hostile and low in warmth. While wealthy individuals elicit feelings of admiration, they also provoke feelings of envy and competition in others. These feelings of envy may provoke hostile behaviour towards the high-status individuals: a recent study showed that in a random-income game, the lower earning individuals paid to reduce the income of the higher earners [31]. As inequity aversion is a strong motivator for punishment [32], this may explain why overcontributors to charity opt to hide their identity.

While the precise motivations behind hidden altruism in this context remain elusive, it seems likely that individuals hide donations when these donations violate established norms of behaviour. Further work, using experimental subjects, could disentangle whether norm deviants avoid derogation, because high donations are seen as competitive or, because high donations are linked to perceived wealth, which can trigger negative feelings associated with inequity aversion. The fact that overcontributors may be punished or ostracized by others—and the apparent desire to hide overly generous behaviour as a consequence—may act as a socially imposed limit to runaway selection for cooperative displays that might otherwise be expected to evolve under the concept of competitive altruism [13]. More theoretical work is now urgently needed to understand how social costs might limit the expression of highly cooperative behaviour.
